# Automatic breast carcinoma detection in histopathological micrographs based on Single Shot Multibox Detector

**DOI:** 10.1016/j.jpi.2022.100147

**Published:** 2022-09-26

**Authors:** Mio Yamaguchi, Tomoaki Sasaki, Kodai Uemura, Yuichiro Tajima, Sho Kato, Kiyoshi Takagi, Yuto Yamazaki, Ryoko Saito-Koyama, Chihiro Inoue, Kurara Kawaguchi, Tomoya Soma, Toshio Miyata, Takashi Suzuki

**Affiliations:** a Department of Pathology and Histotechnology, Graduate School of Medicine, Tohoku University, Sendai, Miyagi 980-8575, Japan; b NEC Solution Innovators, Ltd., Koto-ku, Toyko 136-8627, Japan; c Department of Molecular Medicine and Therapy, Graduate School of Medicine, Tohoku University, Sendai, Miyagi 980-8575, Japan; d Renascience Inc., Chuo-ku, Tokyo 103-0023, Japan; e Department of Anatomic Pathology, Graduate School of Medicine, Tohoku University, Sendai, Miyagi 980-8575, Japan; f Department of Pathology, Sendai Medical Center, Sendai, Miyagi 983-8520, Japan; g NEC Corporation, Minato-ku, Tokyo 108-8001, Japan; h Department of Pathology, Tohoku University Hospital, Sendai, Miyagi 980-8574, Japan

**Keywords:** Breast cancer, Pathology, Artificial intelligence, Deep learning

## Abstract

**Background:**

A diagnosis with histological classification by pathologists is very important for appropriate treatments to improve the prognosis of patients with breast cancer. However, the number of pathologists is limited, and assisting the pathological diagnosis by artificial intelligence becomes very important. Here, we presented an automatic breast lesions detection model using microscopic histopathological images based on a Single Shot Multibox Detector (SSD) for the first time and evaluated its significance in assisting the diagnosis.

**Methods:**

We built the data set and trained the SSD model with 1361 microscopic images and evaluated using 315 images. Pathologists and medical students diagnosed the images with or without the assistance of the model to investigate the significance of our model in assisting the diagnosis.

**Results:**

The model achieved 88.3% and 90.5% diagnostic accuracies in 3-class (benign, non-invasive carcinoma, or invasive carcinoma) or 2-class (benign or malignant) classification tasks, respectively, and the mean intersection over union was 0.59. Medical students achieved a remarkably higher diagnostic accuracy score (average 84.7%) with the assistance of the model compared to those without assistance (average 67.4%). Some people diagnosed images in a short time using the assistance of the model (shorten by average 6.4 min) while others required a longer time (extended by 7.2 min).

**Conclusion:**

We presented the automatic breast lesions detection method at high speed using histopathological micrographs. The present system may conveniently support the histological diagnosis by pathologists in laboratories.

## Introduction

Breast cancer is one of the most common malignancies in females worldwide.^1^ Benign breast disorders include ductal hyperplasia, intraductal papilloma, adenosis, and fibroadenoma.^2^ Conversely, invasive carcinoma accounts for >80% of all breast carcinoma diagnoses, and non-invasive carcinoma account for 10%–20%.^3^^,^^4^ Histological examination of specimens (Hematoxylin and eosin (H&E)-stained tissues) is conventionally used under light microscopy in pathological diagnosis.^5^ Early detection and accurate diagnosis of breast carcinoma with histological classification by pathologists are very important for appropriate treatments to improve the prognosis of patients. However, the number of pathologists is limited and they have too many tasks and are under the stress in many laboratories.^6^, ^7^, ^8^, ^9^ In addition, pathological diagnosis tends to depend on the subjectivity of their experience. Therefore, assisting the pathological diagnosis by objectively double-checking with artificial intelligence (AI) becomes very important.

Several studies about automatic classification for breast pathological images, especially using artificial neural network (ANN) approach, have been previously reported.^10^, ^11^, ^12^, ^13^ K. Kiambe proposed a 2-stage model for 4-class classification (normal, benign, non-invasive carcinoma, and invasive carcinoma) of breast histopathological images, and the model achieved 99.84% accuracy.^14^ Yun Jiang et al. designed a convolutional neural network (CNN) with a small SE-ResNet module to classify BreakHis dataset into 8 subtypes and revealed that the model achieved a 90.66% and 93.81% accuracy.^15^

However, various problems remained to overcome to practically introduce AI into the clinical workflow for pathological diagnosis. Firstly, most tissues include various types of components although many showed the results of classification for whole images and/or heat maps, indicating potential areas where carcinoma cells may be present.^16^, ^17^, ^18^, ^19^, ^20^, ^21^, ^22^, ^23^ Especially, a single result of classification for a whole image by AI leads many users to feel the AI as a black box and be hesitant to use AI.^24^ Thus, providing more information about the processes that lead to the results by showing the areas of each component may be important to make the users feel less inhibited and for practical use as assistance for doctors.

Furthermore, the impact of AI assistance on clinical utility has not been fully investigated while the performance to classify histopathological images has been mainly focused on. Pathological diagnosis is always regarded as the final diagnosis to decide on patient treatments. Moreover, pathological diagnosis is complicated and pathologists diagnose based on their breadth of knowledge and diagnostic experience as stated above. Therefore, completely replacing the role of pathologists with AI may be currently difficult, and confirming the significance of AI as assistance for pathologists and understanding these disadvantages, as well as benefits, is important.

Finally, introducing AI to clinical practice remains a big hurdle regarding cost and trouble. The technology has been developed to digitize an entire glass slide (Whole slide imaging; WSI),^25^ and various AIs to classify WSI have been reported.^19^^,^^26^, ^27^, ^28^ WSI gives various advantages, such as the automated WSI scanner that automatically scans.^29^^,^^30^ However, introducing WSI scanners and digital pathology system and managing huge amounts of digital data need a lot of money. In addition, scanning speed is widely different by machines. For example, while some scanners with high specifications scan 1 slide in 0.5–1.5 min, some WSI scanners take 7–9 min for scanning 1 slide.^31^ Although WSI technology is becoming widespread in clinical practice, actually, using WSI is sometimes difficult in some hospitals.^32^ Thus, proposing the significance of the AI model, which is easy to introduce and simpler to use using micrographs, could be important.

**Fig. 1:**
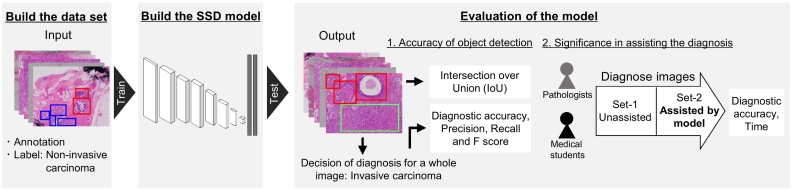
Summary of the present study. We built the data set. The micrographs were taken from glass slides by a pathologist and annotation and image label (benign, non-invasive carcinoma, and invasive carcinoma) were provided for each image. The Single Shot Multibox Detector (SSD) model was trained using 1361 images and evaluated using 315 images. The model performance was evaluated by the intersection over union (IoU) and diagnostic accuracy using detection of the model. To investigate the significance of our model in assisting the diagnosis, 3 pathologists and 5 medical students diagnosed images with or without assistance of the model.

Single Shot Multibox Detector (SSD) is an object detection method that provides detection at high speed in real-time.^33^ The SSD network is relatively simple and can be trained and integrated into systems with comparative ease. The significance of SSD in pathology has not been investigated although it may assist the pathological diagnosis. Therefore, the present study demonstrated an automatic breast lesion detection model using microscopic histopathological images based on SSD for the first time and investigated the effects of the present model in assisting the diagnosis (Fig. 1).

## Materials and methods

### Clinical cases

The H&E-stained^34^ glass slides of histopathological specimens of human breast diseases were obtained from Tohoku University Hospital, Sendai, Japan. All specimens were obtained from patients who had undergone surgical treatment or biopsy at Tohoku University Hospital and had been fixed with 10% formalin neutral buffer solution and embedded in paraffin wax.^35^ Experiments and analyses were performed following the Helsinki declaration, and the research protocol of this study was approved by the Ethics Committee at the Tohoku University Graduate School of Medicine (approval no. 2021-1-1046).

### Datasets and annotations

**Fig. 2:**
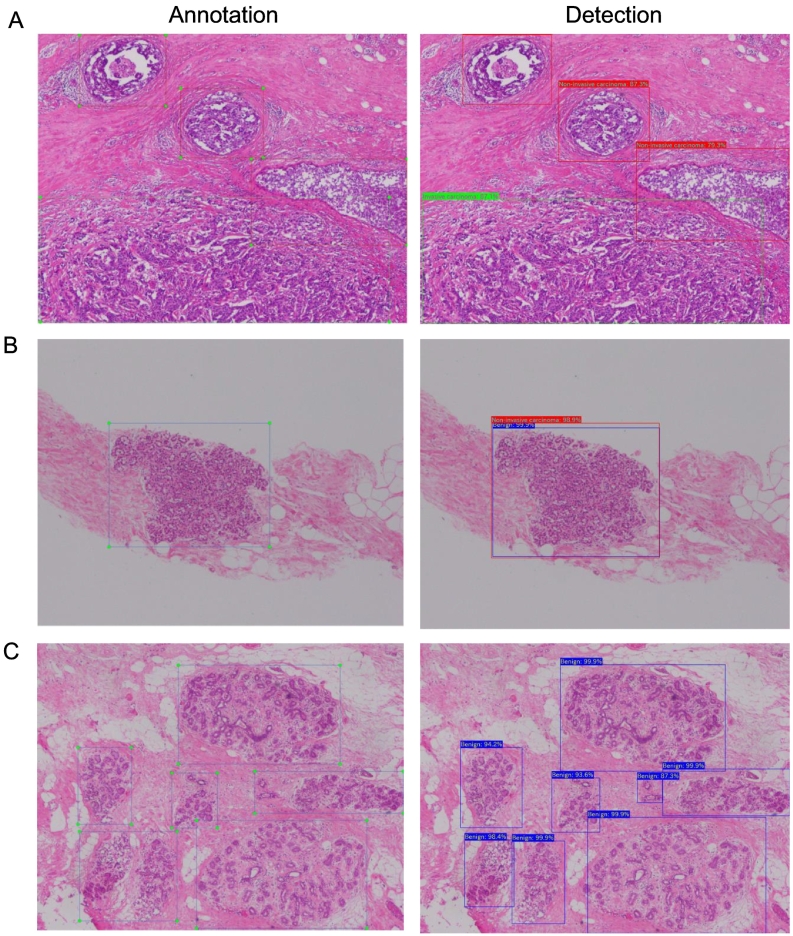
Example images of annotation by pathologists and detection by the trained SSD model. Examples of the annotation (left) and the model detection (right). The blue, red, and green boxses indicated benign, non-invasive carcinoma, and invasive carcinoma, respectively. **A**: The image of accurately detection by the model. **B**: The image that some boxes with different labels were detected in same region. **C**: The image that the model accurately detected the benign area by bounding boxes with different shape against the annotation.

Table 1 showed the dataset summary. The micrographs were taken in PNG formation from glass slides by microscope (Olympus BX53, Olympus Inc.,Tokyo, Japan) with Olympus DP26 digital camera (Olympus) and the software cellSens Dimension (Olympus) by an expert pathologist, especially for breast disorders (annotation pathologist). The size of each image is 2448 × 1920 pixels (72 dpi) at 40× magnification. The images were annotated by a pathologist using the LabelImg v1.8.1 tool (https://github.com/tzutalin/labelImg), drawing bounding boxes around the breast epithelium tissues that conformed with one of these 3 labels (annotation label): invasive carcinoma, non-invasive carcinoma, and benign, including benign lesions and normal breast epithelium tissues in the present study. Representative examples of annotation for various images were shown in Fig. 2A–C.

**Table 1:** The dataset summary.

	Number of pictures				Number of annotations			
	Benign	Non-invasive carcinoma	Invasive carcinoma	Total	Benign	Non-invasive carcinoma	Invasive carcinoma	Total
Training	608	337	416	1361	3516	1485	1294	6295
Test	164	40	111	315	853	154	247	1254
Examination set-1	14	15	14	43	–	–	–	–
Examination set-2	14	15	14	43	–	–	–	–

Then, a single-label diagnosis of the whole image (image label) was provided for each image. Many images include various annotation labels, and an image label was determined following the prioritization, such as invasive carcinoma, non-invasive carcinoma, and benign. Images, including the area of invasive carcinoma, were labeled with “invasive carcinoma” regardless of the presence of non-invasive carcinoma and benign lesions. Images, including components of non-invasive carcinoma without invasive carcinoma cells, were labeled with “non-invasive carcinoma” regardless of the presence of a benign lesion. Images, which did not contain carcinoma cells, were labeled as “benign” in this study. These determination methods followed the same rules as for clinical diagnosis.

### SSD network structure and training

The SSD is an object detection method that accurately detects at high speed in real-time.^33^ SSD is based on a forward propagation CNN network, and, the network produces scores for the presence of each object category in each bounding box and performs adjustments to the boxes to match the object shape for prediction. Furthermore, the network combines predictions from multiple feature maps with various resolutions, thereby processing the objects at diversified sizes. SSD is simple because it sums up all computation in a single network, leading to detection at high speed. Thus, SSD can train and integrate into systems with comparative ease without using machines of too high specifications. We performed 300 000 iterations of the train using 1361 images in the training set and conducted random horizontal flipping for images during training for data augmentation. The test set used 315 images to evaluate the model. An example image of accurate detection by the trained model was shown in Fig. 2A.

### Evaluation measures

This study evaluated the performance of the model in diagnosing each image. The diagnosis of each image was determined following the same rules, as well as giving an image label in building the data set, using the detection results of the trained model. Images were classified as benign, non-invasive carcinoma, or invasive carcinoma in the 3-class task and classified as benign or malignant, including non-invasive carcinoma and invasive carcinoma, in the 2-class task. Images that the model did not detect the lesions were considered as “Benign.” Detection with higher confidence score was used for diagnosis and that with lower confidence score was not used, if some boxes with different labels were detected in the same region (Fig. 2B). Accuracy, recall, precision, and F1-score were used following the formula below to evaluate the diagnosis of images.^36^

$$\text{Accuracy (Diagnostic accuracy)}\left(\%\right)=\begin{array}{l} \text{Accurately classified images}/\text{Total images}\times 100 \end{array}$$

$$\text{Recall}\;\left(\%\right)=\text{Accurately classified target images}/\text{Total target images}\times 100$$

$$\text{Precision}\left(\%\right)=\begin{array}{l} \text{Accurately classified target images}/\text{Total predicted target images}\times 100 \end{array}$$

F1-score = 2* (Recall*Precision)/(Recall + Precision)

Moreover, the intersection over union (IoU) score was used to evaluate the performance of breast lesion detection.^37^ The IoU score was calculated using the below formula.

IoU score = Area of overlap/Area of Union.

### Impact of the SSD model on the diagnosis by pathologists and medical students

We compared the diagnostic accuracy between the trained SSD model, pathologists, and medical school students, and investigated the effects of this model for assisting the diagnosis. A total of 3 experienced pathologists and 5 medical school students who conducted the pathological studies using breast carcinoma tissues participated in the experiments. They were not involved in building a data set. They diagnosed the Examination set-1 and set-2, both with 43 images. The accuracies of the trained model were the same score between Examination set-1 and set-2. First, they diagnosed images in Examination set-1 without the assistance of the trained model. Then, they classified the images in Examination set-2 with the assistance of model detection. The trained model was implemented as a simple tool, which showed the result of object detection and confidence score by inputting an image, and the threshold of detection confidence score could be freely changed in the tool. They were given information about the performance of the trained model, and the selection of the threshold of detection confidence score for display was left to them. All experiments were conducted without time constraints and the time taken for diagnosis of all images in each set was recorded. The diagnosis accuracy and needed time were analyzed between the trained model, pathologists, and medical students, as well as between the model-assisted and unassisted patterns.

### Software, tool, and statistical analysis

All the SSD model experiments were performed using a PC with the following specifications: Intel(R) Core (TM) i5-10300H processor with 16 GB RAM and NVIDIA(R) GeForce RT (TM) 2060 GPU. The models were mounted and trained by TensorFlow, and a tool to simply use this trained model was implemented. IoU score was calculated in python using a shapely package. A student t-test was used to examine differences in the diagnostic accuracies of pathologists and medical school students.

## Results

### Accuracy of diagnosis for images using the SSD model

The SSD model was trained using 1361 images in the training set and evaluated using 315 images in the test set. We investigated the diagnostic accuracy for images using detection by the trained model in the different thresholds of available confidence scores of detected objects. Thresholds of confidence score were changed from 0.1 to 0.9 at intervals of 0.1. The present model achieved a diagnostic accuracy of 85.4% using detection confidence score thresholds of 0.3 and 0.4, as shown in Table 2. The calculated F1-score with recall and precision score showed 89.6% for images labeled with benign (the confidence score threshold of 0.4), 71.0% for non-invasive carcinoma (the confidence score threshold of 0.5), and 88.8% for invasive carcinoma (the confidence score threshold of 0.1). The model achieved a higher diagnostic accuracy of 88.3% using the threshold of detection confidence score when each component showed the highest F1-score (benign for 0.4, non-invasive carcinoma for 0.5, and invasive carcinoma for 0.1 of confidence score thresholds). Furthermore, the trained model showed 90.5% accuracy under the same conditions in a 2-class task to diagnose as benign or malignant (non-invasive carcinoma or invasive carcinoma). Conversely, the model showed a relatively low precision score (68.0%) for the classification of images of non-invasive carcinoma. Additionally, the model sometimes could not detect lesions in the images that showed remarkably weak H&E staining in this study (Supplementary figure).

**Table 2:** Model performance for diagnosis of images in different threshold of confidence score.

			Threshold of detection confidence score.										
			0.1	0.2	0.3	0.4	0.5	0.6	0.7	0.8	0.9	Benign; 0.4 Non-invasive carcinoma; 0.5 Invasive carcinoma; 0.1	
3-class task	Diagnostic accuracy (%)		81.6	83.8	85.4	85.4	84.8	82.9	82.5	80.0	77.8	**88.3**	
	F1-score	Benign	83.9	87.0	88.5	**89.6**	89.1	87.9	87.7	86.5	84.6	Recall	90.9
												Precision	90.9
												F1-score	90.9
		Non-invasive carcinoma	61.3	64.7	68.8	70.2	**71.0**	67.4	68.8	66.0	67.4	Recall	85.0
												Precision	68.0
												F1-score	75.6
		Invasive carcinoma	**88.8**	88.3	88.2	85.4	83.7	81.3	79.8	74.4	68.2	Recall	85.6
												Precision	94.1
												F1-score	89.6
2-class task	Diagnostic accuracy (%)		84.4	86.7	87.9	88.9	88.3	86.7	86.3	84.8	81.9	**90.5**	
	Recall (%)		79.3	84.7	87.9	90.8	91.3	91.0	91.5	91.9	93.5	90.1	
	Precision (%)		91.4	88.1	86.8	85.4	83.4	80.1	78.8	74.8	66.9	90.1	
	F1-score		84.9	86.4	87.3	88.1	87.2	85.2	84.7	82.5	78.0	90.1	

### Performance of object detection by the SSD model

We evaluated the trained model by IoU score, which is a better detection evaluation metric.^37^ The mean IoU of benign detection was 0.52, non-invasive carcinoma was 0.44, and invasive carcinoma was 0.62 when the threshold of detection confidence score for benign was 0.4, non-invasive carcinoma was 0.5, and invasive carcinoma was 0.1, as shown in Table 3. The 3-class average IoU was 0.59.

**Table 3:** Model performance for the breast lesions detection.

Intersection over Union (IoU)^a^			
Benign	Non-invasive carcinoma	Invasive carcinoma	3-class average
0.52 (0.32)	0.44 (0.39)	0.62 (0.34)	**0.59 (0.27)**

a Threshold of detection confidence score; Benign 0.4, Non-invasive carcinoma 0.5, Invasive carcinoma 0.1. Data were presented as mean (STD).

The mean average precision (mAP)^38^ is also useful for evaluating the target localization and detection model, but we did not use it to evaluate this model. Breast lesions could not be divided and counted simply due to the intricacies of these forms and there is a wide range of variations of drawing bounding boxes around them. For example, the model accurately detected the benign area by bounding boxes with different shapes against the annotation boxes as shown in Fig. 2C. Hence, we considered that mAP was not suitable to accurately evaluate this model.

### Comparison of the SSD model with humans and its effects on diagnosis

**Fig. 3:**
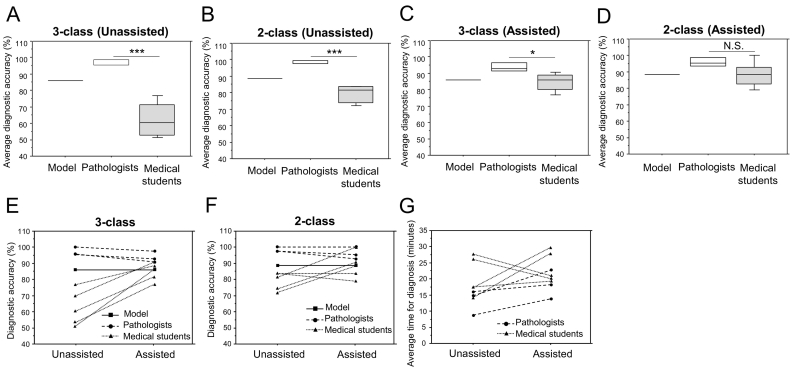
Performance of diagnosis by the SSD model, pathologists, and medical students with or without assistance of the model. **A–D**: The average diagnostic accuracy of the model, pathologists (n = 3), and medical students (n = 5) without assistance (**A, B**) or with assistance (**C, D**) of the model in the 3-class (**A, C**) or 2-class (**B, D**) classification tasks. A student t-test was used to examine differences in the diagnostic accuracies and the data were presented as the mean ± S.D. *P < 0.05, ***P < 0.001 vs model, respectively. N.S.; not significant. **E, F**: The change of each accuracy score with or without assistance of the model in 3-class (**E)** and 2-class (**F)** classification tasks. **G**: The change of the time taken for diagnosis with or without assistance of the model.

We evaluated the trained SSD model as compared with 3 pathologists and 5 medical students using 43 images in Examination set-1. They diagnosed these images without time constraints, and diagnostic accuracy was evaluated. All results were shown in Table 4 and Fig. 3. The model showed higher diagnostic accuracy than medical students while pathologists got remarkably higher accuracy than the model and medical students in both 3-class (Fig. 3A) and 2-class (Fig. 3B) tasks.

**Table 4:** Impact of the SSD model on the diagnosis by pathologists and medical students.

		Examination set-1 (Unassisted)			Examination set-2 (Assisted)		
		Model only^a^	Pathologists (n = 3)	Medical students (n = 5)	Model only^a^	Pathologists (n = 3)	Medical students (n = 5)
3-class task	Diagnostic accuracy (%)	86.0	97.7	67.4	86.0	93.8	84.7
2-class task	Diagnostic accuracy (%)	88.4	98.4	79.1	88.4	96.1	88.4
	Precision (%)	92.9	98.9	78.0	100	96.7	89.6
	Recall (%)	89.7	98.9	96.6	82.8	97.7	93.8
	F1- score	91.2	98.9	86.2	90.6	97.1	91.6
Time taken for diagnosis (min)		–	12.9	20.2	–	17.9	23.3
Time differences between unassisted or assisted tasks (min)		–	–	–	–	+5.0	+3.1

a Threshold of detection confidence score; Benign 0.4, Non-invasive carcinoma 0.5, Invasive carcinoma 0.1. Data of pathologists and medical students were presented as average scores.

We then investigated the significance of the model in assisting the diagnosis by humans by evaluating its effects on diagnostic accuracy and the time taken for diagnosis. They conducted the diagnosis for images in Examination set-2, which contains different 43 images from Examination set-1 but keeps the same composition, with the assistance of the model detection. Medical students showed remarkably higher diagnostic accuracy scores (3-class task: average 84.7%, 2-class task: average 88.4%) with the model assistance compared to that without model assistance (3-class task: average 67.4%, 2-class task: average 79.1%) in both 3-class (Figure 3C) and 2-class (Figure 3D) tasks. Notably, no significant difference was found between the accuracy of pathologists and medical students with the assistance of the model in the 2-class task. Furthermore, some medical students achieved the same or higher accuracy scores than pathologists when using the assistance of the model for diagnosis (Fig. 3E, F). Pathologists achieved a diagnostic accuracy score of more than the 90% average with or without the assistance of the model. Two participants diagnosed images in a short time using the assistance of the model (shorten by average 6.4 min) while six participants required a longer time (extended by average 7.2 min) when we investigated the time taken for diagnosis of all images in each set (Fig. 3G). The average times taken for diagnosis were extended by 5.0 min and 3.1 min in pathologists and medical students, respectively.

## Discussion

The present study firstly demonstrated the SSD model as an object detection method to detect the breast lesions in micrographs of human breast tissues to our best knowledge and evaluated the significance of the model in assisting the diagnosis. Many currently employed AI models that classify breast pathological images were developed using an algorithm, such as ANN, including CNN,^39^, ^40^, ^41^, ^42^ ResNet,^43^^,^^44^ AlexNet,^14^^,^^45^ and Inception-V3,^46^^,^^47^ however, the present model provides more detailed information not only about the classification of images but also location and type of lesion in images, and it could be useful for assisting the pathologists. The 3-class and 2-class classification tasks obtained 88.3% and 90.5% diagnostic accuracies for images, respectively. The diagnosis for images using the model achieved higher accuracy scores than medical students in experiments for model evaluation compared with pathologists and medical students, which would be helpful for pathological diagnosis.

Conversely, pathologists achieved higher accuracies than the model. The mean IoU is 0.59 and there is room for improvement in the performance of the model. There may be a limit to completely annotate and accurately detect lesions by object detection methods using bounding boxes because of the intricacies of these forms. Using other methods, such as semantic segmentation,^48^ with pixel-level annotated images may be efficient if the speed or simplicity of machine specs for implementation is not so important for users. For example, Priego-Torres et al. presented a new framework for carcinoma cell segmentation in breast histopathological images and the estimated segmentation accuracy was 95.62%.^49^ Additionally, new released object detection methods after SSD also may be useful in the aspect of accuracy.^50^

We then investigated the significance of the present model in assisting the diagnosis. Steiner et al evaluated the potential impact of assistance for pathologists by automatic detection of breast carcinoma metastasis in lymph nodes and revealed some benefits of the assistance by AI in pathology.^51^ Very recently, Mantrala et al investigated the concordance rate in breast carcinoma grading as determined by the Nottingham Grading System between AI and pathologists using WSI.^27^ However, the impact of assistance by AI in diagnosing various breast lesions on clinical utility has not been fully investigated. The present study revealed that medical students upregulated accuracy scores when using the assistance of the model and some students achieved the same or higher accuracy scores than pathologists, although the SSD model and medical students showed lower diagnostic accuracies than pathologists. Notably, some medical students showed the best performance when using the assistance of the present model compared with the model only or without the assistance of the model. These results indicated that the present model may be helpful for medical students or pathologists who are not so skilled in breast disorders to accurately diagnose using micrographs, and using AI as assistance has the potential to support pathological diagnosis in laboratories. Additionally, the present model uses micrographs of breast tissues although WSI scanning still takes a lot of time,^32^ and there is a wide range of variation of methods to use. For example, pathologists can take micrographs and quickly confirm the detection of the model only if they hesitate to judge certain components. A microscope is used in routine diagnosis in clinical practice, and the present model may be comparatively easy to introduce and simpler to use at high speed using micrographs. Conversely, the average accuracy of pathologists achieved >90% and could not be changed by the assistance of the model. Further amelioration of the performance of the model can be helpful for pathologists, with a broader range of experience levels, including virtuosic pathologists. Additionally, these experiments were conducted without time constrain and AI may be more effective in stressful situations, such as when many images are needed to be diagnosed under time constrain in clinical practice.

Some medical students achieved high diagnostic accuracy in a short time using the model when pathologists and medical students diagnosed images using the assistance of the model, and this result indicated that assistance by the present model may have the potential to improve both accuracy and efficiency of pathological diagnosis. On the other hands, diagnosing images with assistance took a long time on average than without assistance. The present study used the model by inputting one image at a time. More simple operation methods to use the model could further improve the efficiency. Additionally, further investigation about appropriate conditions in using AI tools will be needed to improve the efficiency of diagnosis for more people. Too much information from AI can lead to confusion among pathologists and increase the workload to process information, thus an appropriate amount of information should be supplied for them. For example, by setting the suitable threshold of confidence score. The relationship between accuracy and efficiency of diagnosis using AI as assistance, when the amount of information from AI is changed, should be explored in the future.

One of the limitations of the present study is the created dataset with specimens obtained from a single institution and it may not represent the heterogeneity of specimens in various facilities. The present model could not detect lesions in images taken by specimens whose hematoxylin staining is weak compared with other specimens. The model should be trained and evaluated using other images taken from specimens in other facilities in the future.

In conclusion, we presented the automatic breast lesions detection method using histopathological micrographs based on SSD, which is an object detection algorithm, for the first time. The model can detect breast lesions in micrographs at high speed with a low cost for the introduction. The model showed 88.3% diagnostic accuracies for images in 3-class classification tasks and medical students improved their diagnosis performance using the assistance of this model. Therefore, the present system conveniently supports the histological diagnosis by pathologists in laboratories.

## Funding

This research did not receive any specific grant from funding agencies in the public, commercial, or not-for-profit sectors.

## Disclosure

The authors report no conflicts of interest in this work.

## Appendix A Supplementary data

Supplementary material Image 1
