# Supplementary material for: Automatic breast carcinoma detection in histopathological micrographs based on Single Shot Multibox Detector
Source: J Pathol Inform. 2022 Sep 26;13:100147. doi: 10.1016/j.jpi.2022.100147 (PMC9577133; doi:10.1016/j.jpi.2022.100147)
Supplement: Supplementary file 1 — Supplementary material [file mmc1.zip › mmc1.pptx]

## Slide 1
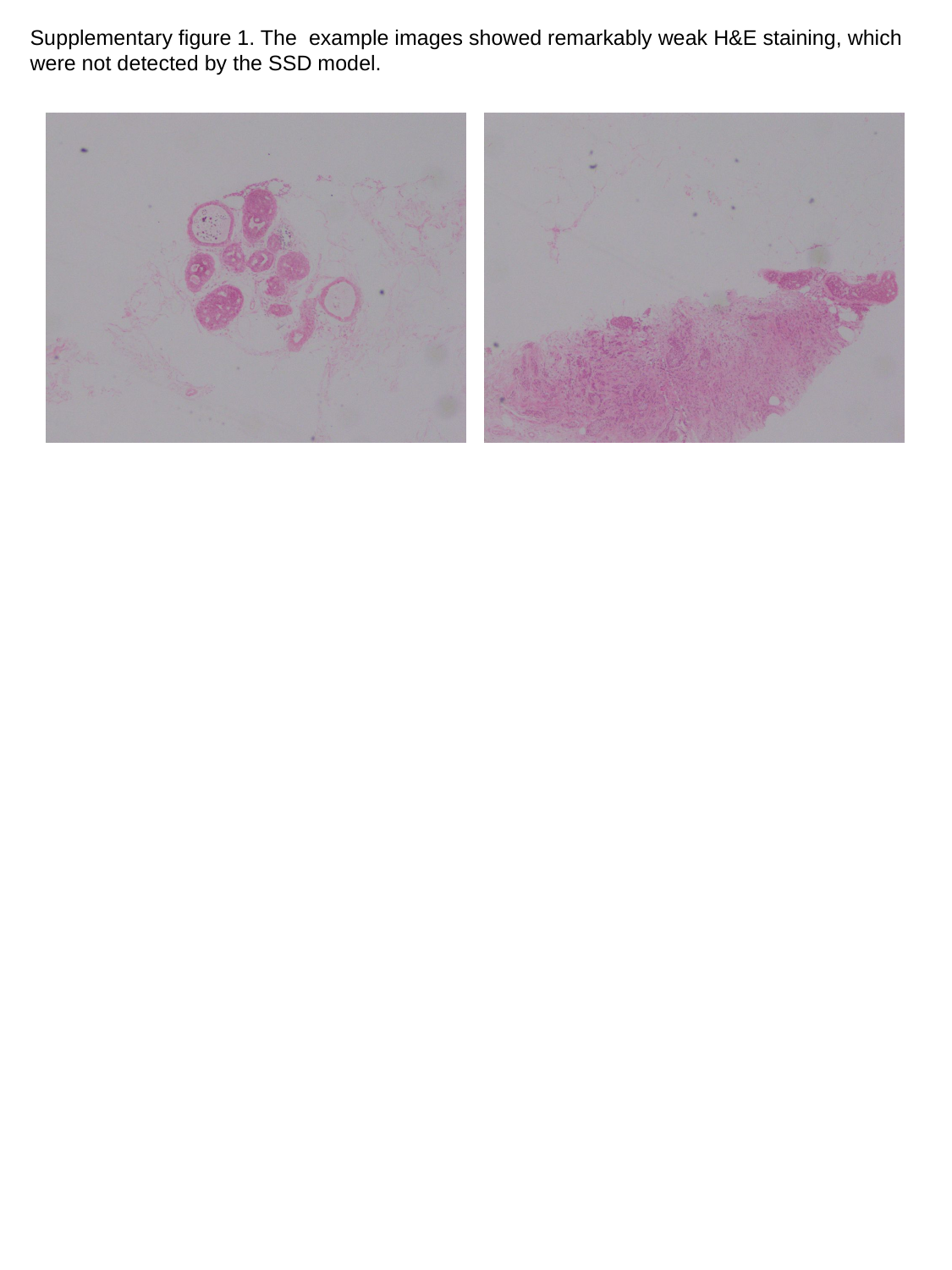

Supplementary figure 1. The example images showed remarkably weak H&E staining, which were not detected by the SSD model.
